# An Efficient Needleless Grasping Suture Technique for Graft Preparation in Anterior Cruciate Ligament Reconstruction

**DOI:** 10.3389/fsurg.2022.863823

**Published:** 2022-05-12

**Authors:** Chaohua Fang, Rongshan Cheng, Jian Jiang, Dimitris Dimitriou, Huizhi Wang, Ziang Jiang, Tsung-Yuan Tsai, Cheng-Kung Cheng

**Affiliations:** ^1^ Department of Sports Medicine, Ningbo No. 6 Hospital, Zhejiang, China; ^2^ School of Biomedical Engineering and Med-X Research Institute, Shanghai Jiao Tong University, Shanghai, China; ^3^ Engineering Research Center of Digital Medicine, Ministry of Education, Shanghai, China; ^4^ Shanghai Key Laboratory of Orthopaedic Implants and Clinical Translation R&D Center of 3D Printing Technology, Department of Orthopaedic Surgery, Shanghai Ninth People’s Hospital, Shanghai Jiao Tong University School of Medicine, Shanghai, China; ^5^ Department of Operating Room, Ningbo No. 6 Hospital, Zhejiang, China; ^6^ Department of Orthopedics, Balgrist University Hospital, Zürich, Switzerland

**Keywords:** needleless grasping suture technique, needle stitching technique, graft preparation time, biomechanical performance, anterior cruciate ligament reconstruction

## Abstract

**Objective:**

Several needleless techniques have been developed to outcome the inherent disadvantages of the traditional needle stitching technique for graft preparation, such as tendon damage through the needle, time consumption, and the potential risk of needlestick injury. The purpose of the present study is to compare the graft preparation time and the biomechanical performance between an efficient needleless technique and the traditional needle stitching technique for graft preparation in anterior cruciate ligament reconstruction (ACLR).

**Methods:**

The time required to perform a complete suture on 20 hamstring tendons during ACLRs was measured. The grafts from one side were prepared using the needle stitching technique. The grafts from the other side used the needleless grasping suture technique. For the second part of the study, 12 fresh-frozen porcine flexor tendons were divided into two groups using two techniques and were mounted in an electric tensile test system. Each group was pretensioned to 100 N to simulate the maximum initial graft tension. The suturing state of sutures and graft (intact and damaged) and the load-elongation curve were recorded for each group. A Student’s *t*-test was used to compare the means of the two groups.

**Results:**

In operation, the needleless grasping suture technique group (19.8 ± 4.8, range: 13.5–32.9 s) was significantly faster (*p* < 0.05) than the needle stitching technique group (52.7 ± 12.7, range: 36.0–87.5 s). The state of sutures in each group was intact. The mean elongation was 11.75 ± 1.38 (range: 9.47–12.99) mm and 10.59 ± 1.02 (range: 9.12–11.76) mm in the needleless stitching technique group and the needle grasping suture technique group, respectively. There was no statistically significant difference in the elongation between the two groups (*p* > 0.05).

**Conclusion:**

The needleless grasping suture technique was a convenient and efficient method for graft preparation in ACLR.

## Introduction

Anterior cruciate ligament (ACL) rupture is one of the most common injuries in sports medicine, especially in young and athletic populations. The estimated number of ACL injuries per year in the United States has increased to over 200,000 annually, and the number of ACL reconstruction (ACLR) performed has reached 120,000 per year (1, 2). For the graft, several choices are available, including autograft [bone-patella tendon-bone (BTB), hamstring tendon (HT)] or allograft tendon, and artificial ligament (3). Among them, the autograft tendon is the most popular graft used. Before the fixation of the autograft tendon, the femoral tunnel and tibial tunnel were created by selecting appropriate surgical techniques, and the graft preparation was performed, including pretension and linkage. The free ends of the tendon are usually weaved for applying tension to the graft during final fixation with an interference screw (4). Therefore, a secure graft preparation technique is essential for this step.

Traditionally, several suturing techniques (5, 6) have been suggested for graft preparation, such as the Krackow stitch, the baseball stitch, and the whipstitch. In order to outcome the inherent disadvantages of the traditional needle stitching technique, such as tendon damage through the needle, time consumption, and the potential risk of needlestick injury, several needleless techniques have been developed, including the modified Prusik knot, Wittstein suture loop, rolling hitch, modified finger-trap, and modified rolling hitch (7). Recently, a newly released device SpeedTrapTM (DePuy-Mitek, Raynham, MA, USA) (8) completely eliminated the use of needles, and it also can create a tubular tendon configuration over a 3 cm expanse of the tendon. This study (8) also confirmed that the overall performance of the SpeedTrapTM technique in terms of preparation speed, fixation security, biomechanical strength, and resultant tissue trauma was far superior to that of the other five different graft fixation techniques (OrthoCord Krackow stitch, FiberWire Krackow stitch, FiberLoop, WhipKnot, and Loop-in-loop).

The previous study (8) showed that the SpeedTrap technique provided a shorter graft preparation time than the Krackow stitching technique and a biomechanical performance similar to that of this totechnique. However, the SpeedTrap technique required expensive equipment, corresponding high medical expenses, and complex teaching operations. To confer advantages for graft preparation without expensive equipment and complex teaching operations, we developed an efficient needleless grasping suture technique. We hypothesized that the needleless grasping suture technique might provide a shorter graft preparation time than the needle stitching technique and a biomechanical performance similar to that ofthis technique.

## Methods

### Surgical Time for Graft Preparation

The clinical study was approved by the Ethics Committee (Approve number: L2021100). The patients were treated with arthroscopic ACLR. The inclusion criteria of the recruited patients were (i) they should have suffered from acute trauma within the previous 6 weeks, (ii) should have had symptoms of knee instability and other clinical evidence of ACL insufficiency verified by positive Lachman tests, (iii) MRI scans should reveal no other ligament injury except for ACL rupture, with or without accompanied meniscus injury, (iv) patients should be in good health, (v) patients should not have undergone previous ipsilateral knee joint surgery, and (vi) patients should not have been treated with medications known to affect bone metabolism. During ACLR, the time for the graft preparation of 20 HTs using the needleless grasping and the needle stitching technique (Whipstitch) (9) was recorded using a digital chronometer. A braided absorbable suture with a needle was used for graft suturing, with the thread material consisting of copolymer (polyglactin-910) of glycolide and lactide (1–0 VICRYL PLUS®, Ethicon, Somerville, NJ, USA). The grafts were sutured using two techniques starting at 20 mm from the distal free end of the grafts.

### *In vitro* Mechanical Testing

**Figure 1:**
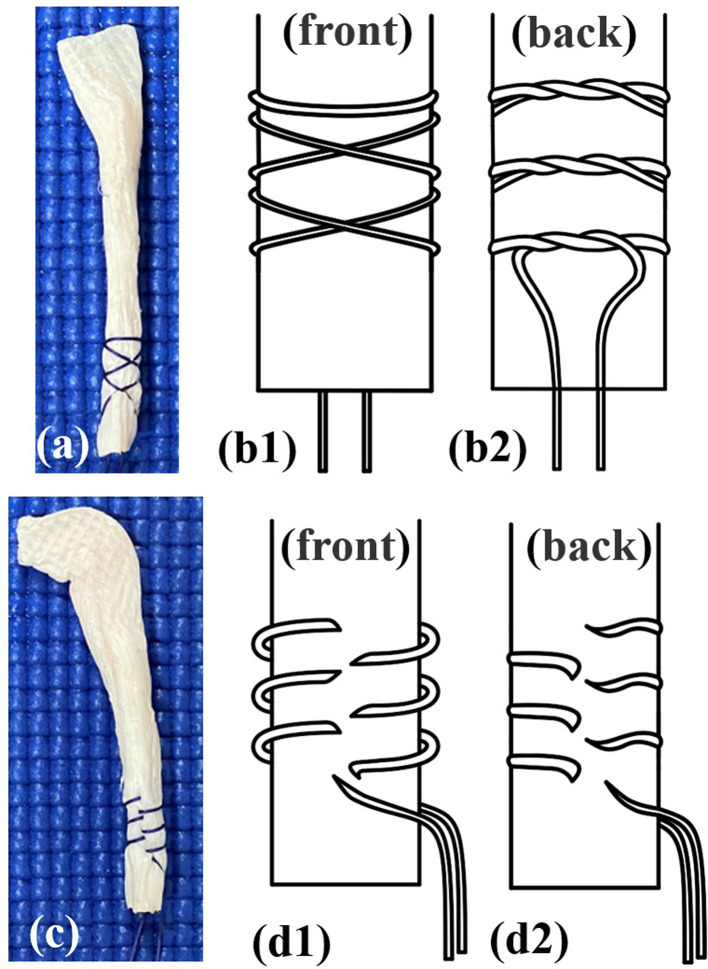
Two suture techniques are investigated: a photograph (**A**) and diagram (**B1**,**B2**) of the needleless grasping suture technique group, and a photograph (**C**) and diagram (**D1**,**D2**) of the needle stitching technique group. The needleless grasping suture technique group is started by wrapping the suture around the porcine flexor tendon, and the working end of the suture is then crossed over the other end of the suture. The graft preparation is completed with a hitch by making a turn around the porcine flexor tendon.

**Figure 2:**
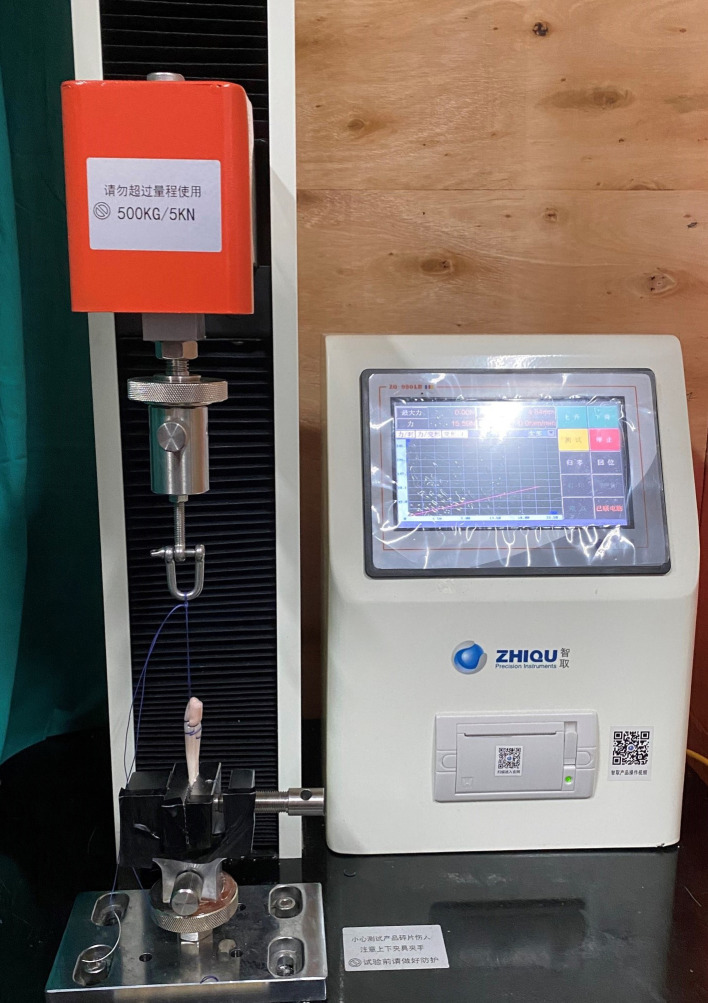
The graft with the needle stitching technique is placed in an electric tensile test system before the pretension test.

**Figure 3:**
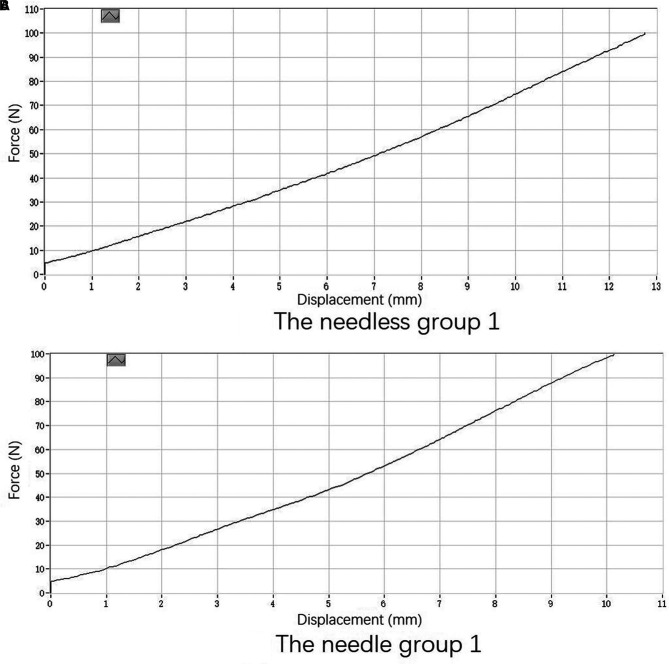
One typical load-elongation curve is shown. (**A**) The needleless group 1 and (**B**) the needle group 1.

For the second part of the study, according to a *post hoc* power analysis with 83% power (an alpha error of 0.05, a total sample size = 12, and an effect size = 0.93), 12 fresh-frozen porcine flexor tendons were collected from a local abattoir when the pigs were slaughtered (Approve number: 202101260). The porcine flexor tendons divided into two groups were cut to the same length (10 cm) for mechanical testing (Figure 1). The weave was finished starting at 20 mm from the distal free end of the grafts with needleless grasping or Whipstitch, respectively. The mechanical testing was performed from each group using an electric tensile test system (ZQ-990, ZHIQU Precision Instrument Co., Ltd, China). The speed of the loading was set to 20 mm/min (6). First, each group was pretensioned to 100 N to simulate the clinical surgeon’s operation to remove the slack of the ligaments before testing the grafts. Second, 100 N tensile loading was used to simulate the maximum initial graft tension (10). Under the 100 N tensile loading kept for 1 min, the suturing state of the sutures and graft (intact or damaged) was observed during the experiment, and the load-elongation curve of the grafts was recorded for each group (Figure 2). One typical load-elongation curve is shown in Figure 3.

### Statistical Analysis

All data were normally distributed and expressed as mean ± standard deviation. A Student’s *t*-test was used to compare the means of two groups. Statistical analysis was performed using SPSS 24.0 (SPSS Inc., Chicago, IL, USA). The significance level (*α*) was set at 0.05. A *post hoc* power analysis (an alpha error of 0.05, a total sample size = 12, and an effect size = 0.93) was conducted with G*power software 3.1.9 (Franz Faul, Christian-Albrechts-Universität Kiel, Kiel, Germany) according to the results of the graft preparation time and the elongation of grafts.

## Results

The *post hoc* power analyses indicated a calculated 83% power to detect the effects on the graft preparation time and the elongation of grafts.

**Figure 4:**
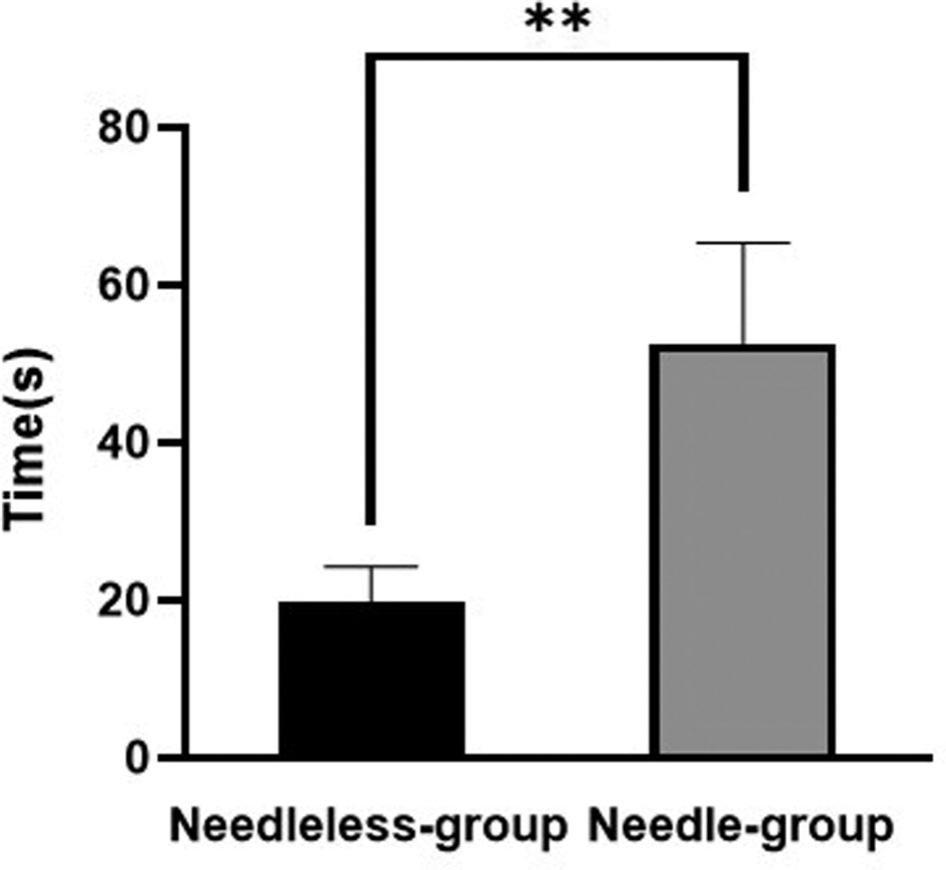
The mean graft preparation time in the needle stitching technique group (needle group) and the needleless grasping suture technique group (needleless group). **p < 0.05.

The mean graft preparation time was 19.8 ± 4.4 s (range: 13.5–32.9 s) and 52.7 ± 12.7 s (range: 36.0–87.5 s) in the needleless grasping suture technique group and the needle stitching technique group, respectively. Therefore, the needleless grasping suture technique groups were faster than the needle stitching technique groups (*p* < 0.05) (Figure 4).

**Figure 5:**
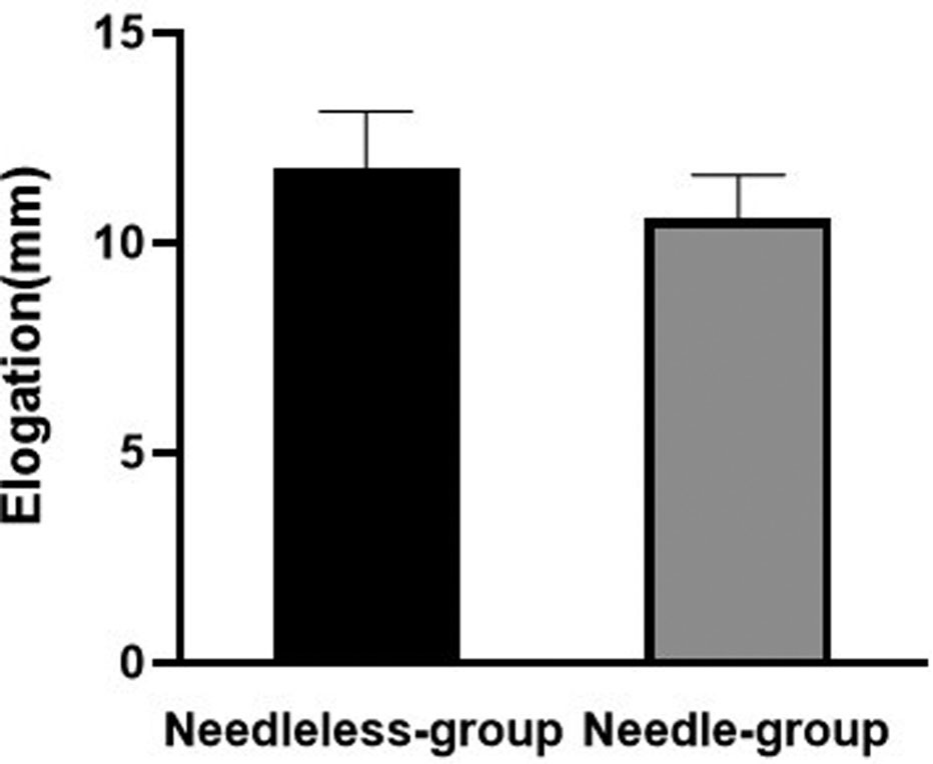
The mean load elongation in the needle group and the needleless group.

The suturing state of the sutures and grafts in each group was intact. The mean elongation was 11.8 ± 1.4 mm (range: 9.5–13.0mm) and 10.6 ± 1.0 mm (range: 9.1–11.8 mm) in the needleless stitching and the needle grasping suture technique, respectively. There was no statistically significant difference in the elongation between the two groups under the loading (*p* > 0.05) (Figure 5).

## Discussion

The principal finding of the present study was that the time consumed for the graft preparation of the newly introduced needleless technique was significantly shorter than that of Whipstitch, but with similar elongation within 100 N. The results of the present study might suggest that the presented needleless technique might be a reliable alternative without the inherent disadvantages of the traditional needle stitching technique for graft preparation, such as tendon damage through the needle, time consumption, and the potential risk of needlestick injury.

Graft preparation is crucial for the success of ACLR, and it may vary by the type and linkage method of the graft. For example, the preparation of BTB mainly focuses on the shape and diameter of the bonebolt (11). For the tendon, the end may be weaved or sutured together, according to the necessity to fold, the fixation device, or the technique (12). Among them, a single folded tendon is most simple and used most extensively. When Endobutton™ (Smith & Nephew, MA, USA) was used as a fixation device at the femoral side, the ends of the tendon were weaved and folded after passing through the loop (13). After drawing the tendon into the tibial and femoral tunnel, the ends were tightened outside the tibial tunnel and fixed. Usually, the tendon can be kept in tension by tightening the weaved sutures with bare hands or a device such as a tensioner of the INTRAFIX® ADVANCE Tibial Fastener System. The tensile force was no more than 100 N (14, 15). After fixing the tendon at the tibial side, the extra free end and sutures need to be excised. Therefore, the weave of the tendon ends seemed just to take effect of an easy pass through the tunnel and as strings to keep tension. Traditionally, the ends were weaved using the needle suture (16). However, it was time-consuming, laborious, and required skill. Considering the role of the weave, a less time-consuming, simpler, but a firmly fixed method can be used. Sasho et al. (16) tested the mechanical strength of tendon graft samples by employing different suture techniques of joining the two free ends. They found that the ultimate load was the highest in the double zigzag locking loop stitch group (558.7 ± 47.6 N), but a statistically significant difference was not observed among the groups. Another *in vitro* biomechanical test compared several methods (6), including the modified finger-trap suture technique, Krackow stitch, locking SpeedWhip stitch, and nonlocking SpeedWhip stitch. The results showed that, although the smallest percent elongation was different, the load to failure (377–396 N) and cross-sectional area were not significantly different across all the suture groups. These results indicate that the failure load is much larger than the tensile force needed during operation, because the over-tensioned grafts may lead to a limitation of the range of motion and damage the articular surface. Kim et al. (17) compared the effect of subjective clinical results, anterior laxity, and knee extensor strength in the ACLR from an initial tendon graft tension force including 8, 12, or 15 kg. They found no significant differences. Furthermore, a recent study (15) investigated the effect of the femorotibial positional relationship exerted by initial graft tension. The results showed that a high initial graft tension (maximum manual force) resulted in an external rotation of the tibia against the femur just after anatomical ACLR. Still, low initial graft tension (80 N at full knee extension) did not change the femorotibial rotational relationship (15). Consequently, we simulated the tension exerted on the tendon before fixation with interference screw and compared only the elongation curve by 100 N tensile loading in the current study. Our results showed no significant difference between the needleless technique and the needle weave, which confirmed the safety and reliability of the needleless technique.

There is convincing evidence that operation time is an essential factor for risk and complications after isolated ACLR or knee arthroscopy. Even marginal increases in operative time are associated with an increased risk of adverse events such as deep vein thrombosis, surgical site infections, sepsis, an extended length of stay, and readmissions (18, 19). In addition, it can reduce anesthesia time and potentially reduce aesthesia-associated complications in patients. We recorded and compared the time needed for graft preparation with these two techniques during real operation. The results were in accordance with the previous study (7) performed on the animal experiment with flexor profundus tendons harvested from fresh pig hind-leg trotters. The time consumed by the needleless technique is obviously less. In the current study, the weave process was completed by a proficiency scrub nurse. Considering the laborious technique required for needle weave, the time consumed may be longer for unskilled staff. The time saved by graft preparation can shorten the operation time of ACLR and may reduce the risk of adverse events after ACLR.

Furthermore, this needleless technique did not require stitches, which represented other advantages. First, it increased the safety of healthcare workers. Non-requirement of the stitch needle reduced the risk of needlestick injury to the scrub nurse, surgeon, or assistant, so as to avoid occupational exposure to the common sources of infection such as hepatitis B virus, hepatitis C virus, and human immunodeficiency virus (20). Second, it reduced the economic burden. According to information provided by the manufacturers of sutures, the cost of the needle was much higher than that of the suture. For the thread attached suture needle, the needle accounts for much of the cost rather than the thread. As a result, the needle technique saved the cost of the needle. Considering the amount of ligament reconstruction, the economic burden may be greatly reduced if this technique is adopted extensively.

Our study has several limitations. First, only one kind of needle stitching technique and suture material was used in our study, including real operation and mechanical tests. Second, we adopted the whipstitch and chose the braided absorbable suture with the needle (1–0 VICRYL PLUS®, Ethicon, Somerville, NJ, USA) according to our routine clinical practice. Third, due to the expensive equipment (SpeedTrap**^TM^**) and lack of financial support, the SpeedTrap**^TM^** technique was not included in this study for difference comparison.

In conclusion, the needleless grasping suture technique was a convenient and efficient method for graft preparation following ACLR without expensive equipment and complex teaching operations. The newly introduced needleless grasping suture technique demonstrated a shorter graft preparation time but pretensioning of the ligament compared with the needle whipstitch technique, suggesting that the presented needleless technique might be a reliable alternative.

## Data Availability

The original contributions presented in the study are included in the article/Supplementary Material; further inquiries can be directed to the corresponding author/s.
